# Comparison of VISUMAX 800 and VISUMAX 500 Femtosecond Laser Systems for Myopia: A Systematic Review and Meta-Analysis

**DOI:** 10.3390/jcm15145517

**Published:** 2026-07-14

**Authors:** Qi Wan, Ran Wei, Li Chen, Ke Ma

**Affiliations:** Department of Ophthalmology, West China Hospital of Sichuan University, Chengdu 610044, China

**Keywords:** VISUMAX 800, VISUMAX 500, SMILE, myopia, meta-analysis, systematic review

## Abstract

**Objectives:** The VISUMAX 800 (Carl Zeiss Meditec) is the second-generation femtosecond laser for small incision lenticule extraction (SMILE), featuring faster pulse rates, automated cyclotorsion compensation (OcuLign), and automated centration (CentraLign) versus the VISUMAX 500. This systematic review and meta-analysis compared their clinical outcomes in myopia correction. **Methods:** Following PRISMA 2020 guidelines, we searched PubMed, EMBASE, and Web of Science through March 2026 for studies comparing the two platforms in myopia or myopic astigmatism with extractable data. Primary outcomes were predictability (SE ± 0.50 D) and astigmatism (CYL ≤ 0.50 D). Secondary outcomes included UDVA ≥ 20/20, safety (CDVA loss ≥ 1 line), R^2^ values, surgically induced astigmatism, axis alignment (±5°), and higher-order aberrations. Risk of bias was assessed using the ROBINS-I tool for all included studies, as no randomized controlled trials were available. Publication bias was evaluated via funnel plots, Egger’s test, and Begg’s test, with appropriate caution noted regarding the limited number of studies. Sensitivity analysis used the leave-one-out method. **Results:** Nine studies (1672 eyes: 646 VISUMAX 800, 1026 VISUMAX 500) were included. For SE ± 0.50 D, the pooled risk ratio (RR) was 1.065 (95% CI: 0.997–1.137, *p* = 0.061) with substantial heterogeneity (I^2^ = 64.7%, *p* = 0.004). For CYL ± 0.50 D (eight studies, 1585 eyes), the pooled RR was 1.022 (95% CI: 0.978–1.068, *p* = 0.333, I^2^ = 51.7%). Astigmatism axis within ±5° significantly favored VISUMAX 800 (RR = 1.157, 95% CI: 1.071–1.250, *p* = 0.0002, I^2^ = 0%). No statistically significant differences were observed for UDVA ≥ 20/20, safety, SEQ R^2^, cylinder R^2^, TIA, SIA, total HOAs, spherical aberration, or coma RMS. Publication bias tests showed no significant asymmetry for the primary outcomes, though these tests have limited power with fewer than 10 studies. ROBINS-I assessments classified most studies as having “serious” risk of bias due to their non-randomized designs. **Conclusions:** Both platforms yield comparable predictability, safety, and visual outcomes. VISUMAX 800 offers superior astigmatism axis alignment, likely due to automated compensation and centration. The borderline SE predictability warrants further randomized investigation

## 1. Introduction

Myopia has reached epidemic proportions globally, with the prevalence continuing to rise across both developed and developing nations. Refractive surgery has become an increasingly important modality for the correction of myopia, offering patients the possibility of spectacle and contact lens independence. Among the various refractive surgical techniques available, small-incision lenticule extraction (SMILE), also referred to as keratorefractive lenticule extraction (KLEx), has emerged as a minimally invasive, flap-free procedure that preserves corneal biomechanical integrity while providing predictable and stable refractive outcomes [1].

The VISUMAX femtosecond laser platform (Carl Zeiss Meditec, Jena, Germany) is one of the two predominant laser systems used to perform SMILE procedures worldwide. The first generation of this platform, VISUMAX 500, received regulatory approval and has been widely adopted since its introduction, with a substantial body of evidence supporting its safety, efficacy, and predictability for myopia correction. The laser operates at a pulse frequency of 500 kHz, with a typical refractive lenticule creation time of approximately 25–30 s [2,3].

More recently, the second-generation platform, VISUMAX 800, has been introduced with a combination of hardware and software upgrades that together represent a broader technological platform rather than a simple laser system iteration. The hardware advancements include an increased pulse frequency of 2000 kHz, which reduces the refractive lenticule creation time to less than 10 s—a feature expected to decrease the risk of intraoperative suction loss and improve overall surgical efficiency. On the software side, VISUMAX 800 incorporates two computer-assisted navigation aids: OcuLign for automated cyclotorsion correction and CentraLign for optimized centration control. These software features are designed to address two recognized limitations of the first-generation platform—imprecise astigmatism axis alignment and suboptimal centration—which can compromise visual quality and refractive predictability. Specifically, OcuLign targets improved astigmatism axis alignment by compensating for cyclotorsional eye rotation during docking, while CentraLign aims to improve corneal centration, which may favorably influence higher-order aberration (HOA) induction and overall optical quality [4].

It is important to recognize that both the VISUMAX 500 and VISUMAX 800 platforms already achieve high levels of safety, efficacy, and predictability in myopia correction, with most studies reporting over 90% of eyes within ±0.50 D of the intended correction. The clinically relevant question, therefore, is not whether one platform is broadly superior, but whether the specific advantages of VISUMAX 800—including faster laser time, improved cyclotorsion compensation, and automated centration—translate into domain-specific improvements that reach a meaningful clinical threshold. For example, a modest improvement in overall spherical equivalent predictability may be less impactful than a substantial improvement in astigmatism axis accuracy for patients with moderate-to-high astigmatism. This distinction is important for interpreting the comparative evidence.

Despite the growing body of literature on VISUMAX 800 [5,6,7,8,9,10,11,12,13], no systematic review or meta-analysis has yet synthesized the comparative evidence between the two platforms. The primary clinical question of this study is whether the technical upgrades of VISUMAX 800 translate into clinically meaningful improvements in predictability and astigmatic correction compared to VISUMAX 500, while secondarily evaluating safety, visual quality, and surgical efficiency outcomes. The central focus is on refractive control—particularly whether the VISUMAX 800 achieves superior spherical equivalent and cylinder predictability—given that these outcomes are most directly relevant to surgeons deciding between platforms. To address this gap, we conducted a systematic review and meta-analysis of all comparative studies reporting outcomes after SMILE using VISUMAX 800 versus VISUMAX 500 for the correction of myopia and myopic astigmatism.

## 2. Methods

### 2.1. Protocol, Registration and Eligibility Criteria

This systematic review and meta-analysis was conducted in accordance with the Preferred Reporting Items for Systematic Reviews and Meta-Analyses 2020 (PRISMA 2020) statement [14] and the Cochrane Handbook for Systematic Reviews of Interventions [15]. This systematic review was pre-registered PROSPERO: CRD420261407686.

Studies meeting the following PICOS criteria were included:

Population (P): Adult patients (≥18 years) with myopia or myopic astigmatism undergoing primary SMILE. Studies limited to hyperopia, post-refractive surgery enhancement, or cataract surgery were excluded.

Intervention (I): SMILE performed using the VISUMAX 800 femtosecond laser (Carl Zeiss Meditec AG, Jena, Germany).

Comparator (C): SMILE performed using the VISUMAX 500 femtosecond laser (Carl Zeiss Meditec AG, Jena, Germany).

Outcomes (O): Primary outcomes: (1) proportion of eyes within ±0.50 D of intended spherical equivalent (SE ± 0.50 D); (2) proportion of eyes within ≤0.50 D of intended cylinder (CYL ≤ 0.50 D). Secondary outcomes: (3) UDVA ≥ 20/20; (4) CDVA loss ≥ 1 line; (5) SEQ R^2^; (6) cylinder R^2^; (7) TIA; (8) SIA; (9) astigmatism axis alignment ±5°; (10) total HOAs; (11) spherical aberration; (12) coma RMS.

Study design (S): Comparative studies (RCTs, prospective or retrospective cohorts, case–control) with separate outcome data for VISUMAX 800 and VISUMAX 500 groups. Single-arm studies, reviews, editorials, and case reports were excluded.

### 2.2. Information Sources and Search Strategy

A systematic literature search was conducted across three electronic databases: PubMed (MEDLINE), EMBASE, and Web of Science. The search was last performed on 26 March 2026, with no date restrictions applied. The search strategy combined Medical Subject Headings (MeSH) terms and free-text keywords: (“VISUMAX 800” OR “VisuMax 800” OR “SMILE Pro” OR “second-generation”) AND (“VISUMAX 500” OR “VisuMax 500” OR “SMILE” OR “KLEx” OR “keratorefractive lenticule extraction” OR “femtosecond laser platform” OR “femtosecond laser system”) AND (“myopia” OR “myopic astigmatism” OR “refractive surgery”). The search was supplemented by manually scanning reference lists of included studies and relevant review articles. To ensure adequate search sensitivity—particularly given that VISUMAX 800 is a recent technology—we employed broad free-text terms including “KLEx”, “lenticule extraction”, and “femtosecond laser platform” alongside the specific device names. The search strategy was validated by re-running with the expanded terms, confirming that no additional eligible studies beyond those identified were retrieved. All 2026 publications were verified as available online ahead of print by 26 March 2026.

### 2.3. Study Selection

**Figure 1:**
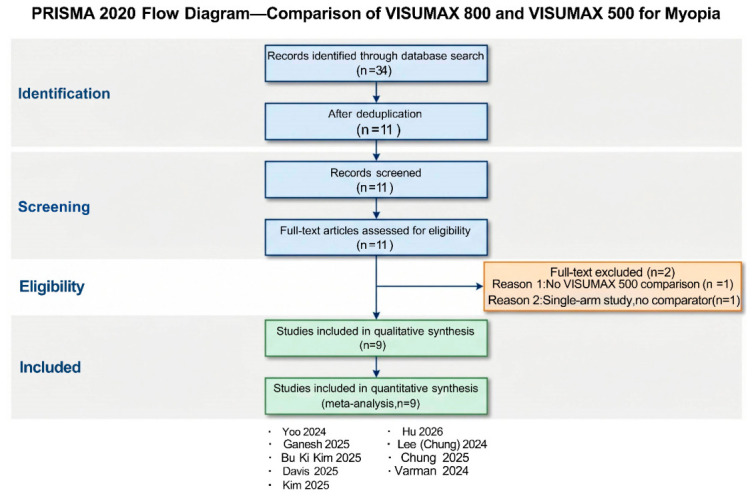
PRISMA 2020 flow diagram. Shows the number of records identified, screened, assessed for eligibility, and included at each stage of the systematic review. Nine comparative studies were included in both qualitative and quantitative synthesis [5,6,7,8,9,10,11,12,13].

Two reviewers independently screened titles and abstracts of all identified records. Full-text articles of potentially eligible studies were then assessed for final inclusion. Discrepancies were resolved by discussion and consensus. Studies were excluded at the full-text stage with reasons documented (see PRISMA flow diagram, Figure 1).

### 2.4. Data Extraction

Data were extracted independently by two reviewers using a standardized data extraction form. Extracted data included: study characteristics (author, year, PMID, journal, country, study design, sample size, follow-up duration), patient demographics (mean age, sex distribution, preoperative refraction), surgical parameters (laser settings, nomogram adjustments, use of cyclotorsion compensation or centration assistance), and all relevant clinical outcomes at each follow-up time point. For studies reporting multiple time points, the 3-month or closest equivalent postoperative visit was prioritized for analysis; where 6-month data were available for the primary outcomes, these were preferentially extracted. All visual acuity and refractive outcomes were extracted as monocular eye-level data.

Discrepancies in data extraction were resolved by referring to the original study and reaching consensus. Corresponding authors were not contacted for additional data due to the publicly available nature of all included studies via PubMed Central.

### 2.5. Risk of Bias Assessment

The risk of bias in individual studies was assessed using the revised Cochrane Risk of Bias Tool for Randomized Trials (ROB2) [16] for RCTs and the Risk of Bias in Non-Randomized Studies of Interventions (ROBINS-I) [17] for non-randomized comparative studies. Due to the absence of RCTs in this field, ROBINS-I was effectively applied to all included studies. Each study was assessed across five domains: confounding, selection of participants, classification of interventions, deviations from intended interventions, missing data, measurement of outcomes, and selection of reported results. Studies were classified as having low, moderate, serious, or critical risk of bias.

### 2.6. Statistical Analysis

#### 2.6.1. Data Synthesis

For binary outcomes (SE ± 0.50 D, CYL ≤ 0.50 D, UDVA ≥ 20/20, CDVA loss ≥ 1 line, axis ±5°), the risk ratio (RR) was calculated using the Mantel–Haenszel random-effects model; risk difference (RD) was supplementary. For continuous outcomes (R^2^, TIA, SIA, HOAs), the mean difference (MD) was calculated using DerSimonian–Laird random-effects models. For paired bilateral eye studies (where one eye received VISUMAX 800 and the contralateral eye received VISUMAX 500), paired *t*-tests or repeated-measures analyses were preferred; for unmatched cohort studies, independent samples comparisons were used. All studies were pooled using a random-effects model, which accommodates between-study design variability through the between-study variance component (τ^2^).

#### 2.6.2. Heterogeneity Assessment

Quantified using I^2^ (25%/50%/75% = low/moderate/high) and Cochran’s Q (*p* < 0.10 significant); tau^2^ was estimated via REML [18].

#### 2.6.3. Sensitivity Analyses

Sensitivity analysis for the primary outcome (SE ± 0.50 D) was performed using the leave-one-out method, iteratively removing one study at a time to assess the robustness of the pooled estimate, with an additional post hoc subgroup analysis stratified by follow-up duration (≤3 months vs. >6 months).

#### 2.6.4. Publication Bias

Publication bias was assessed visually using funnel plots and statistically using Egger’s regression asymmetry test [19] and Begg’s rank correlation test [20] for the primary outcomes. *p* < 0.10 was considered indicative of significant publication bias. Additionally, the trim-and-fill method [21] was applied to adjust for potential publication bias in the primary outcome.

#### 2.6.5. Statistical Software

All meta-analyses were performed using R software (version 4.5.1, R Foundation for Statistical Computing, Vienna, Austria) with the meta (version 7.0-0), metafor (version 4.0-0), and robvis (version 0.3.0) packages. A two-sided *p*-value < 0.05 was considered statistically significant unless otherwise specified.

## 3. Results

### 3.1. Study Selection

The PRISMA 2020 flow diagram is presented in Figure 1 and the checklist is afforded in Table S1. The database search yielded 11 records after deduplication. After title and abstract screening, 11 records were assessed for full-text eligibility. Two studies were excluded: one [22] lacked a VISUMAX 500 comparator arm, and one [23] was a single-arm case series without comparative data. Nine comparative studies [5,6,7,8,9,10,11,12,13] met all inclusion criteria and were included in the final qualitative and quantitative synthesis.

### 3.2. Study Characteristics

Table 1 summarizes the characteristics of the nine included studies. Studies were published between 2024 and 2026, conducted across seven countries (India, South Korea, Taiwan, China, Australia, United States), and enrolled a total of 1672 eyes (646 VISUMAX 800, 1026 VISUMAX 500). Follow-up ranged from 1 to 6 months postoperatively. Study designs included prospective bilateral contralateral studies (*n* = 1), retrospective comparative cohorts (*n* = 6), and ambispective cohort studies (*n* = 2). None of the included studies were randomized controlled trials.

### 3.3. Risk of Bias Assessment

**Figure 2:**
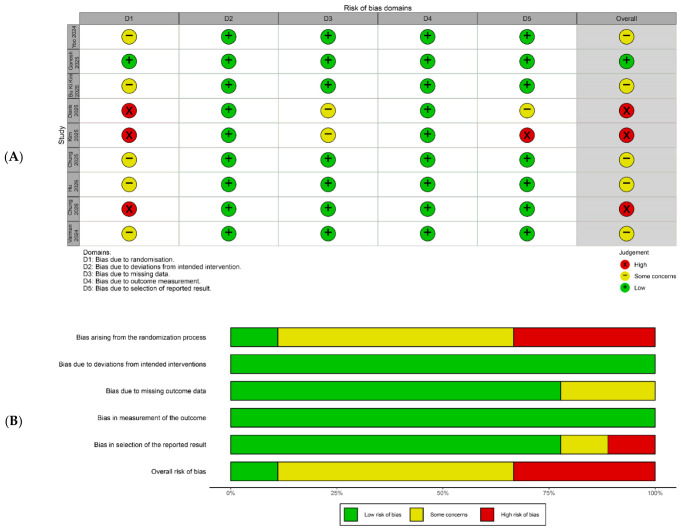
Risk of bias assessments. (**A**) Traffic light plot showing domain-level judgments for each included study. (**B**) Summary plot showing the proportion of studies at each risk-of-bias level; ROBINS-I was applied for non-randomized studies [5,6,7,8,9,10,11,12,13].

The ROBINS-I risk of bias assessment is presented in Figure 2. Overall, six studies were rated as having “some concerns” (primarily due to non-randomized design and potential confounding), and three studies (Davis 2025 [6], Kim 2025 [10], Chung 2025 [5]) were rated as “high” risk of bias due to significant baseline group imbalances, retrospective design without matching, or incomplete outcome reporting. No studies were rated as “low” risk of bias overall. The predominance of non-randomized, retrospective comparative designs represents a fundamental limitation of the available literature in this field.

### 3.4. Primary Outcomes

#### 3.4.1. Predictability: SE Within ±0.50 D

**Figure 3:**
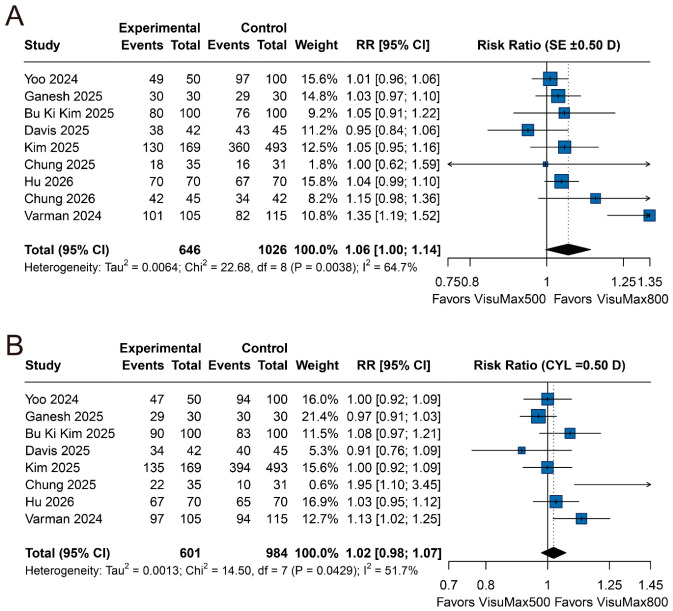
Forest plots for predictability. (**A**) SE within ±0.50 D: RR = 1.07 (95% CI: 1.00–1.14), *p* = 0.061, I^2^ = 64.7%. (**B**) CYL ≤ 0.50 D: RR = 1.02 (0.98–1.07), *p* = 0.333, I^2^ = 51.7%. VISUMAX 500 as control [5,6,7,8,9,10,11,12,13].

Nine studies (1672 eyes) reported the proportion of eyes achieving SE within ±0.50 D. Under the random-effects model, VISUMAX 800 showed a numerically higher likelihood of achieving this predictability endpoint that did not reach statistical significance (RR = 1.065, 95% CI: 0.997–1.137, *p* = 0.061). Substantial between-study heterogeneity was observed (I^2^ = 64.7%, Q = 22.68, df = 8, *p* = 0.0038), which indicates that the pooled estimate should be interpreted with considerable caution, as the true effect may vary substantially across populations and settings. Varman 2024 [12] showed the largest effect size (RR = 1.349), suggesting that this study may be a key driver of heterogeneity. The pooled risk difference for SE ± 0.50 D was RD = 0.056 (95% CI: −0.002 to 0.113, *p* = 0.058). The forest plot is presented in Figure 3A.

#### 3.4.2. Predictability: CYL Within ≤0.50 D

Eight studies (1585 eyes) reported the proportion of eyes with astigmatism within ≤0.50 D. No significant difference was observed between VISUMAX 800 and VISUMAX 500 (RR = 1.022, 95% CI: 0.978–1.068, *p* = 0.333). Moderate heterogeneity was present (I^2^ = 51.7%, Q = 14.50, df = 7, *p* = 0.043). The forest plot is presented in Figure 3B.

### 3.5. Secondary Outcomes

#### 3.5.1. Astigmatism Axis Alignment

**Figure 4:**
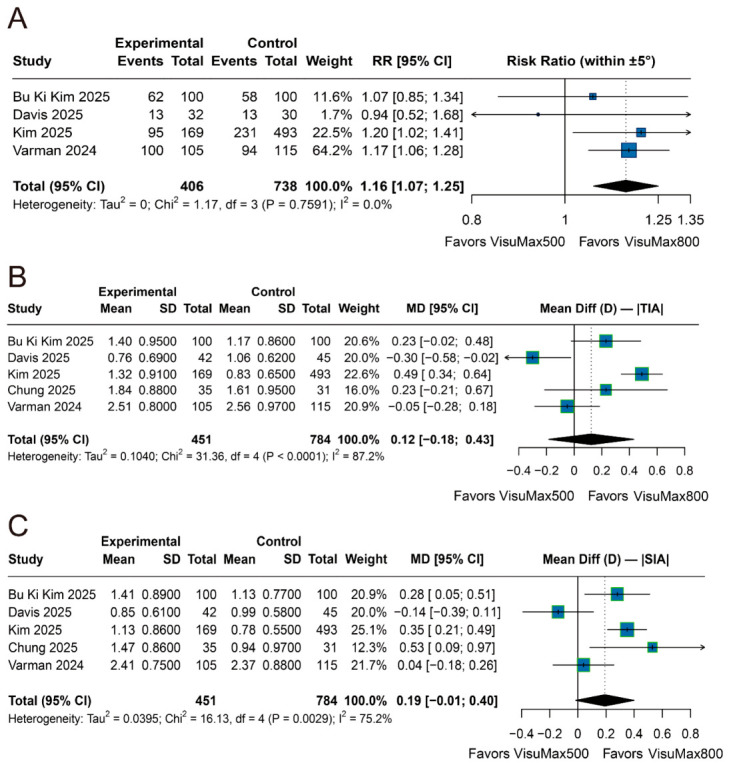
Astigmatism outcomes. (**A**) Axis within ±5°: RR = 1.16 (1.07–1.25), *p* = 0.0002, I^2^ = 0%. (**B**) TIA: MD = 0.12 D (−0.18 to 0.43), I^2^ = 87.2%. (**C**) SIA: MD = 0.19 D (−0.02 to 0.40), I^2^ = 75.2%. Positive MD or RR > 1 favors VISUMAX 800 [5,6,9,10,12].

Four studies (1144 eyes) reported the proportion of eyes with astigmatism axis within ±5° of the intended axis. VISUMAX 800 showed a statistically significant and clinically meaningful advantage (RR = 1.157, 95% CI: 1.071–1.250, *p* = 0.0002), with no between-study heterogeneity (I^2^ = 0%). This translates to a 15.7% relative increase in the likelihood of achieving ±5° axis alignment with VISUMAX 800. The forest plot is presented in Figure 4A. This is the strongest and most consistent finding across all secondary outcomes, likely attributable to the OcuLign cyclotorsion compensation system available on VISUMAX 800.

#### 3.5.2. Astigmatic Correction Metrics

TIA was reported in five studies (1235 eyes). No significant difference was observed between platforms (MD = +0.124 D, 95% CI: −0.184 to +0.433, *p* = 0.430), though high heterogeneity was noted (I^2^ = 87.2%) (Figure 4B). SIA was reported in the same five studies; VISUMAX 800 showed a numerically higher SIA absolute value that did not reach statistical significance (MD = +0.192 D, 95% CI: −0.015 to +0.400, *p* = 0.069), with I^2^ = 75.2% (Figure 4C).

#### 3.5.3. Visual Acuity and Safety

**Figure 5:**
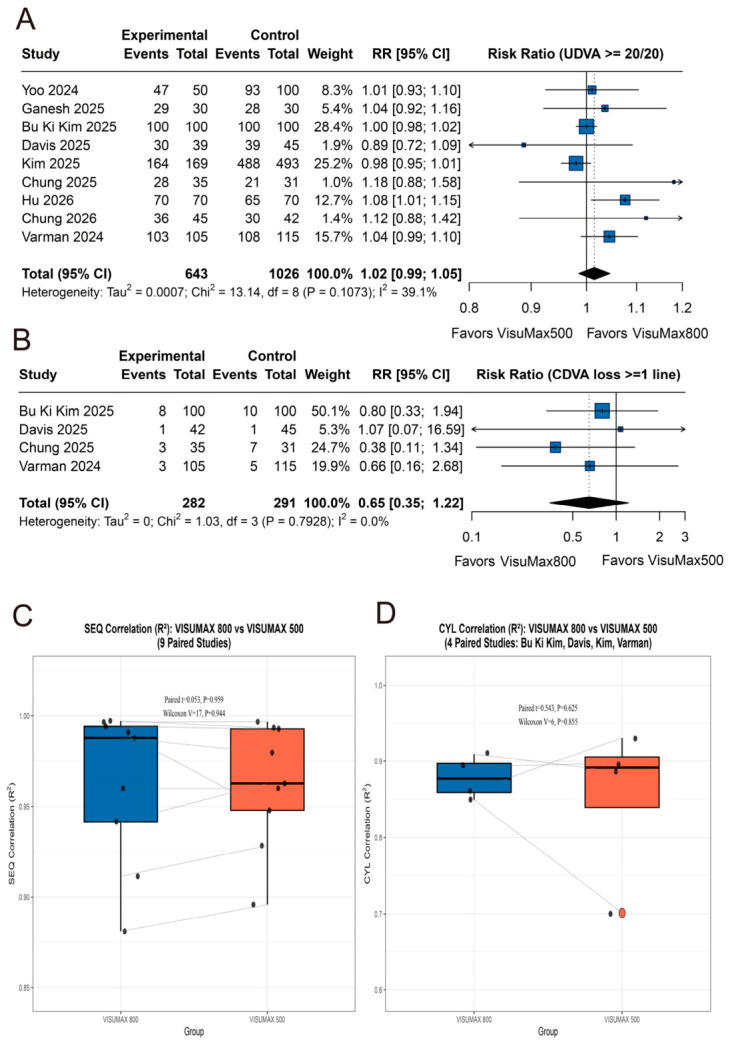
Visual acuity, safety, and correlation. (**A**) UDVA ≥ 20/20: RR = 1.02 (0.99–1.05), I^2^ = 39.1%. (**B**) CDVA loss ≥ 1 line: RR = 0.65 (0.35–1.22), I^2^ = 0%. (**C**) SEQ R^2^: *p* = 0.959. (**D**) CYL R^2^: *p* = 0.625. No significant differences [5,6,7,8,9,10,11,12,13].

UDVA ≥ 20/20 was reported in all nine studies (1669 eyes). The pooled RR was 1.015 (95% CI: 0.986–1.046, *p* = 0.322, I^2^ = 39.1%), indicating no significant difference between platforms. CDVA loss ≥ 1 line was reported in four studies (573 eyes); the pooled RR was 0.650 (95% CI: 0.347–1.217, *p* = 0.178, I^2^ = 0%), also non-significant but directionally favoring VISUMAX 800. Forest plots are presented in (Figure 5A,B).

#### 3.5.4. SEQ and CYL Correlation Coefficients (R^2^)

For SEQ R^2^ (nine paired or comparative groups), the paired analysis confirmed no significant difference (paired *t*-test, *p* = 0.959). For CYL R^2^ (4 studies, 806 eyes), also non-significant. Box plots visualizing these comparisons are presented in Figure 5C,D.

#### 3.5.5. Higher-Order Aberrations

**Figure 6:**
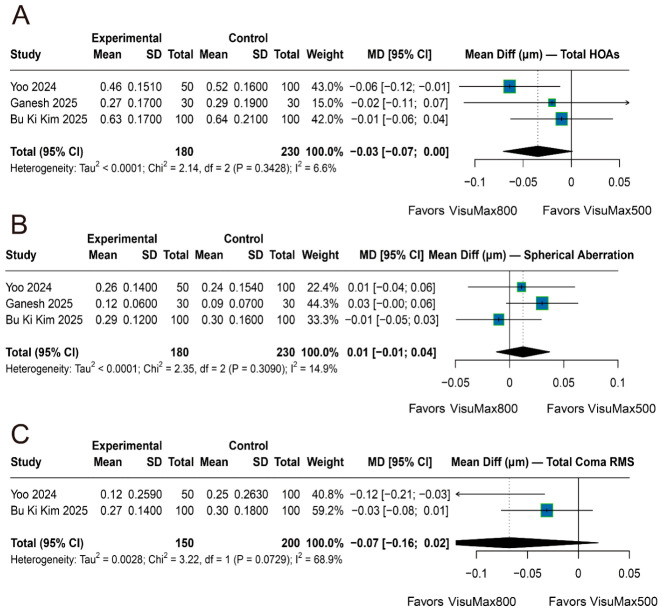
Higher-order aberrations. (**A**) Total HOAs: MD = −0.04 μm (−0.07 to 0.00), *p* = 0.059, I^2^ = 6.6%. (**B**) Spherical aberration: MD = 0.01 μm (−0.01 to 0.04), I^2^ = 14.9%. (**C**) Coma RMS: MD = −0.07 μm (−0.16 to 0.02), I^2^ = 68.9%. Negative MD favors VISUMAX 800 [7,9,13].

Total HOA induction was reported in three studies (410 eyes). VISUMAX 800 showed a trend toward lower HOA induction (MD = −0.035 μm, 95% CI: −0.071 to +0.001, *p* = 0.059) that did not reach statistical significance and with low heterogeneity (I^2^ = 6.6%). Spherical aberration induction was reported in the same three studies (MD = +0.012 μm, 95% CI: −0.012 to +0.037, *p* = 0.322, I^2^ = 14.9%). Coma RMS was reported in only two studies (350 eyes); the MD was −0.068 μm (95% CI: −0.155 to +0.019, *p* = 0.127), with moderate heterogeneity (I^2^ = 68.9%). Forest plots are presented in Figure 6A–C. These analyses should be considered exploratory, as they are based on a limited number of studies (k = 2–3) and did not reach statistical significance for most individual aberration metrics.

### 3.6. Publication Bias

Funnel plots for SE ± 0.50 D and CYL ≤ 0.50 D are presented in Figure S1. Visual inspection suggested mild asymmetry for SE ± 0.50 D, with Varman 2024 [12] appearing as an outlier. Egger’s regression test for SE ± 0.50 D yielded t = 1.93 (df = 7, *p* = 0.094), and Begg’s rank correlation test yielded z = 0.73 (*p* = 0.465), suggesting no statistically significant publication bias at the *p* < 0.10 threshold. For CYL ≤ 0.50 D, Egger’s test yielded t = 0.71 (df = 6, *p* = 0.505) and Begg’s test yielded z = 0.56 (*p* = 0.577), indicating no significant publication bias.

The trim-and-fill analysis for SE ± 0.50 D did not identify any missing studies to be imputed, and the adjusted pooled RR remained 1.065 (95% CI: 0.997–1.137), consistent with the primary analysis. The publication bias assessment summary is presented in Table 2. However, given the limited number of included studies (n = 9), these tests have reduced statistical power to detect funnel plot asymmetry, and the absence of statistically significant results does not definitively exclude publication bias. Visual inspection of the funnel plot for SE ± 0.50 D suggests possible asymmetry, particularly driven by the outlying effect of Varman 2024 [12].

### 3.7. Sensitivity Analysis

The leave-one-out sensitivity analysis for SE ± 0.50 D is presented in Table 3. The pooled RR ranged from 1.030 (after excluding Varman 2024 [12]) to 1.098 (after excluding Hu 2026 [8]), with *p*-values ranging from 0.061 to 0.003. Notably, the pooled estimate became non-significant upon removal of Varman 2024 (RR = 1.030, *p* = 0.083) [12]. Conversely, excluding Hu 2026 [8] increased the pooled effect (RR = 1.098, *p* = 0.003), suggesting this study was suppressing the overall effect size. These findings highlight the influence of individual studies on the pooled estimate and underscore the need for cautious interpretation given the high residual heterogeneity.

To further explore potential sources of heterogeneity, subgroup analyses (≤3 months vs. ≥6 months) were performed in Table 4. The RR for the ≤3 months subgroup was 1.083 (95% CI: 0.987–1.189, *p* = 0.093, I^2^ = 70.2%), while the ≥6 months subgroup yielded an RR of 1.028 (95% CI: 0.992–1.066, *p* = 0.127, I^2^ = 0.0%). The between-group difference was not statistically significant (Q_between = 1.73, df = 1, *p* = 0.188), indicating that follow-up duration did not significantly modify the comparative effect of VISUMAX 800 versus 500 on achieving SE ± 0.50 D. Notably, the ≥6 months subgroup comprised only two studies (Yoo 2024 [13] and Hu 2026 [8]) with zero heterogeneity, whereas the ≤3 months subgroup exhibited substantial heterogeneity (I^2^ = 70.2%), suggesting greater variability among short-term follow-up studies.

## 4. Discussion

This systematic review and meta-analysis synthesized data from nine comparative studies encompassing 1672 eyes (646 VISUMAX 800, 1026 VISUMAX 500). The principal findings can be summarized as follows: 1. Predictability (SE ± 0.50 D): VISUMAX 800 showed a marginally higher likelihood of achieving SE ± 0.50 D (RR = 1.065), but this difference was not statistically significant (*p* = 0.061) and accompanied by substantial heterogeneity (I^2^ = 64.7%). The clinical magnitude of this difference is modest. 2. Astigmatism correction (CYL ≤ 0.50 D): No significant difference was observed between platforms (RR = 1.022, *p* = 0.333), with moderate heterogeneity. 3. Astigmatism axis alignment (±5°): VISUMAX 800 demonstrated a statistically significant and consistent advantage (RR = 1.157, *p* = 0.0002, I^2^ = 0%). This represents the most robust finding of the meta-analysis, likely driven by the OcuLign cyclotorsion compensation and CentraLign centration assistance unique to the VISUMAX 800 platform. The clinical relevance of the 15.7% relative improvement in astigmatism axis alignment should be interpreted in the context of real-world refractive surgery practice. In SMILE without cyclotorsion compensation, axis misalignment of even 5–10° can result in undercorrection of up to 17–35% of the cylindrical component. For a patient with −2.00 D cylinder, a 5° axis error reduces effective correction to approximately −1.83 D, while a 10° error reduces it to approximately −1.70 D. The axis alignment advantage is therefore most clinically meaningful when: (1) the preoperative cylinder is moderate-to-high (≥1.50 D); (2) the astigmatism axis is oblique; and (3) patients exhibit significant intraoperative cyclotorsion (≥3–5°). (4) Safety and visual acuity: No significant differences were observed for UDVA ≥ 20/20, CDVA loss ≥ 1 line, SEQ R^2^, or CYL R^2^. (5) HOA induction: A borderline significant trend toward lower total HOA induction with VISUMAX 800 was observed (*p* = 0.059, I^2^ = 6.6%), suggesting a potential optical quality advantage, though the clinical significance of a ~0.035 μm difference is uncertain.

Based on the findings of this meta-analysis, several patient subgroups are expected to derive greater benefit from VISUMAX 800: (1) patients with moderate-to-high astigmatism (≥1.50 D); (2) eyes with oblique astigmatism axes; (3) patients with significant intraoperative cyclotorsion (≥3–5°); (4) patients with difficult fixation (high anxiety, nystagmus, poor cooperation), who benefit from the shorter lenticule creation time; and (5) surgeons with less experience in manual cyclotorsion compensation.

The findings of this meta-analysis are broadly consistent with the individual included studies. Ganesh et al. [7] reported comparable visual and refractive outcomes between platforms at 3 months, with the primary advantage of VISUMAX 800 being reduced surgical time and higher patient preference. Bu Ki Kim et al. [9] found comparable UDVA, CDVA, and HOA outcomes, with significantly better optic zone centration and lower vertical coma induction with VISUMAX 800. Hu et al. [8] reported comparable visual outcomes but significantly lower opaque bubble layer (OBL) proportions and better lenticular surface regularity with VISUMAX 800. Varman et al. [12] reported a notably higher proportion of eyes within ±0.50 D in the VISUMAX 800 group (96% vs. 71%), attributable to the use of OcuLign and CentraLign in that cohort.

The variation in effect sizes across studies—particularly the outlying result of Varman 2024 [12]—likely reflects differences in surgical technique, use of cyclotorsion compensation, patient populations (Varman 2024 included only high astigmatism ≥1.5 D), and study design. The high heterogeneity observed for TIA (I^2^ = 87.2%) and SIA (I^2^ = 75.2%) merits careful consideration of its potential sources. First, variation in surgeon-specific nomograms is a likely major contributor. Second, differences in surgeon experience and surgical technique affect SIA by altering the biomechanical response of the cornea. Third, baseline patient characteristics differed among studies. Fourth, variability in measurement devices (Scheimpflug tomography vs. Placido-based topography vs. autorefraction) may introduce systematic differences. Fifth, differential use of OcuLign cyclotorsion compensation between arms may inflate SIA heterogeneity. Future studies should standardize nomograms, report surgeon experience levels, and use uniform measurement protocols.

The VISUMAX 800 platform’s primary mechanical improvement is the reduction in lenticule creation time from approximately 28–30 s (VISUMAX 500) to approximately 10 s. This shorter suction time is associated with a lower risk of suction loss [24,25,26,27], which may be particularly advantageous in patients with less cooperative intraoperative fixation. Importantly, the advantages observed with VISUMAX 800 should not be attributed solely to the increased laser pulse frequency. The platform’s improvements reflect a combination of factors, including: (1) the OcuLign cyclotorsion compensation system; (2) the CentraLign automated centration assistance; (3) evolving surgeon nomograms that benefit from accumulated clinical experience; and (4) increasing surgeon familiarity with the platform over time. Disentangling the independent contribution of each factor is not possible from the available comparative studies and represents an important direction for future research [28,29].

For researchers, this meta-analysis highlights the urgent need for well-designed, prospective comparative studies—ideally randomized—with standardized outcome reporting, longer follow-up (≥12 months), and explicit reporting of surgical parameters (nomogram use, cyclotorsion compensation settings). The marked heterogeneity across studies for several outcomes suggests that future individual patient data meta-analyses may help identify subgroups of patients who benefit differentially from each platform.

This meta-analysis followed a pre-registered protocol, applied PRISMA 2020 methodology, included only comparative studies with extractable numerical data, assessed risk of bias systematically, evaluated publication bias, and performed sensitivity analyses. The total sample size of 1672 eyes provides reasonable statistical power for the primary outcomes. However, there are several limitations.

Limitations of the included primary studies: First, the absence of randomized controlled trials means all included studies have non-randomized designs subject to confounding and selection bias. The VISUMAX 800 group in several studies received OcuLign and CentraLign assistance not available in the VISUMAX 500 group, confounding the interpretation of platform-specific vs. software-specific effects. Second, follow-up duration was limited to 3 months in most studies. Third, several studies used eye-level analysis without adjusting for inter-eye correlation. Fourth, some studies may have overlapping patient cohorts.

Limitations of this systematic review: First, substantial heterogeneity was observed for SE ± 0.50 D (I^2^ = 64.7%), and the leave-one-out analysis confirmed the pooled estimate was not robust. Second, several secondary outcomes were based on only 2–3 studies. Third, some outcomes required approximation during pooling due to different metrics. Fourth, CDVA loss data were sparse. Fifth, publication bias tests have limited reliability with fewer than 10 studies. Sixth, mixed follow-up time points (3-month prioritized, 6-month when available) may introduce heterogeneity. Seventh, eye-level analysis in refractive studies may underestimate variance. Eighth, different nomograms across centers influence predictability independent of platform. Ninth, most studies were retrospective, introducing selection bias. Tenth, chronological confounding (improvement in technique and nomograms over time) cannot be excluded.

## 5. Conclusions

This systematic review and meta-analysis of nine comparative studies (1672 eyes) found that VISUMAX 800 and VISUMAX 500 provide comparable predictability and safety for myopia correction. VISUMAX 800 demonstrates a statistically significant advantage in astigmatism axis alignment within ±5° (*p* = 0.0002), likely attributable to its integrated cyclotorsion compensation and centration assistance software. The SE ± 0.50 D predictability finding did not reach statistical significance (*p* = 0.061) and warrants larger, well-designed comparative studies. Future research should focus on prospective, randomized designs with standardized outcome measures, longer follow-up, and explicit reporting of surgical parameters to better characterize the clinical advantages of next-generation femtosecond laser platforms for SMILE.

## Figures and Tables

**Table 1:** Characteristics of included studies.

Study	PMID	Author	Year	Journal	Country	Design	N (800/500)	Follow-Up
Yoo 2024 [13]	38796537	Yoo TK	2024	Sci Rep	South Korea	Retro matched 1:2	50/100	6 months
Ganesh 2025 [7]	39783817	Ganesh S	2025	J Refract Surg	India	Prospective bilateral	30/30	3 months
Bu Ki Kim 2025 [9]	40664759	Kim BK	2025	Sci Rep	South Korea	Retro comparative	100/100	3 months
Davis 2025 [6]	40926937	Davis JC	2025	Cureus	USA	Retro comparative	42/45	3 months
Kim 2025 [10]	41095669	Kim JW	2025	Diagnostics	South Korea	Retro case–control	169/493	3 months
Lee (Chung) 2024 [11]	41535335/39333584	Lee CY	2024	Sci Rep	Taiwan	Retro cohort	35/31	3 months
Hu 2026 [8]	41742367	Hu J	2026	J Cataract Refract Surg	China	Prospective comparative	70/70	6 months
Chung 2025 [5]	41488012	Chung JP	2025	Clin Ophthalmol	Australia	Retro comparative	45/42	1 month
Varman 2024 [12]	39635258	Varman A	2024	Clin Ophthalmol	India	Amnispective cohort	105/115	3 months

**Table 2:** Publication bias assessment.

Test	Primary Outcome	Statistic	*p*-Value	Interpretation
Egger’s regression	SE ± 0.50 D	t = 1.93, df = 7	0.094	No significant bias
Begg’s rank correlation	SE ± 0.50 D	z = 0.73	0.465	No significant bias
Egger’s regression	CYL ≤ 0.50 D	t = 0.71, df = 6	0.505	No significant bias
Begg’s rank correlation	CYL ≤ 0.50 D	z = 0.56	0.577	No significant bias
Trim-and-fill	SE ± 0.50 D	k added = 0	N/A	No adjustment needed

**Table 3:** Leave-one-out sensitivity analysis for SE ± 0.50 D.

Excluded Study	PMID	k Remaining	RR	95% CI	*p*-Value
None (all 9)	—	9	1.065	0.997–1.137	0.061
Yoo 2024 [13]	38796537	8	1.071	0.995–1.151	0.072
Ganesh 2025 [7]	39783817	8	1.069	0.998–1.145	0.055
Bu Ki Kim 2025 [9]	40664759	8	1.067	0.993–1.147	0.075
Davis 2025 [6]	40926937	8	1.078	1.008–1.153	0.028
Kim 2025 [10]	41095669	8	1.063	0.982–1.150	0.124
Lee (Chung) 2024 [11]	39333584	8	1.069	0.999–1.143	0.052
Hu 2026 [8]	41742367	8	1.098	1.032–1.168	0.003
Chung 2025 [5]	41488012	8	1.061	0.993–1.133	0.075
Varman 2024 [12]	39635258	8	1.030	0.996–1.065	0.083

**Table 4:** Subgroup sensitivity analysis for SE ± 0.50 D.

Subgroup	k	Eyes	RR	CI_Lower	CI_Upper	*p*_Value	I2
≤3 months	7	1382	1.0833	0.9869	1.1891	0.0925	0.70%
>6 months	2	290	1.0283	0.9921	1.0659	0.1271	0.00%
Overall	9	1672	1.0646	0.9972	1.1365	0.0607	0.60%
Subgroup difference			NA	NA	NA	0.1882	42.30%

## Data Availability

The datasets used in the current study are available from the corresponding author on reasonable request.

## Supplementary Materials

The following supporting information can be downloaded at: https://www.mdpi.com/article/10.3390/jcm15145517/s1, Figure S1: Funnel plots for SE ± 0.50 D and CYL ≤ 0.50 D; Table S1: PRISMA_2020_checklist.
