# Supplementary material for: Comparison of VISUMAX 800 and VISUMAX 500 Femtosecond Laser Systems for Myopia: A Systematic Review and Meta-Analysis
Source: J Clin Med. 2026 Jul 14;15(14):5517. doi: 10.3390/jcm15145517 (PMC13412861; doi:10.3390/jcm15145517)
Supplement: Supplementary file 1 [file jcm-15-05517-s001.zip › Table S1-PRISMA_2020_checklist_FILLED.pdf]

PRISMA 2020 Checklist

Section and Topic	Item #	Checklist item	Location where item is reported
**TITLE**			
Title	1	Identify the report as a systematic review.	Title page, line 1: "Comparison of VISUMAX 800 and VISUMAX 500 Femtosecond Laser Systems for Myopia: A Systematic Review and Meta-Analysis"
**ABSTRACT**			
Abstract	2	See the PRISMA 2020 for Abstracts checklist.	Abstract, lines [5]–[9] (Purpose, Methods, Results, Conclusions)
**INTRODUCTION**			
Rationale	3	Describe the rationale for the review in the context of existing knowledge.	Introduction, paragraph [11]–[14]
Objectives	4	Provide an explicit statement of the objective(s) or question(s) the review addresses.	Introduction, paragraph [14], final sentence: "we conducted a systematic review and meta-analysis of all comparative studies reporting outcomes after SMILE using VISUMAX 800 versus VISUMAX 500"
**METHODS**			
Eligibility criteria	5	Specify the inclusion and exclusion criteria for the review and how studies were grouped for the syntheses.	Methods, Section 2.2 (lines [19]–[24]), PICOS criteria for Population, Intervention, Comparator, Outcomes, Study design

PRISMA 2020 Checklist

Section and Topic	Item #	Checklist item	Location where item is reported
Information sources	6	Specify all databases, registers, websites, organisations, reference lists and other sources searched or consulted to identify studies. Specify the date when each source was last searched or consulted.	Methods, Section 2.3 (line [26]): PubMed (MEDLINE), EMBASE, and Web of Science. Last searched March 26, 2026. No date restrictions. Supplementary hand-searching of reference lists.
Search strategy	7	Present the full search strategies for all databases, registers and websites, including any filters and limits used.	Methods, Section 2.3 (line [26]): Search strategy provided: ("VISUMAX 800" OR "VisuMax 800" OR "SMILE Pro") AND ("VISUMAX 500" OR "VisuMax 500" OR "SMILE") AND ("myopia" OR "myopic astigmatism" OR "refractive surgery")
Selection process	8	Specify the methods used to decide whether a study met the inclusion criteria of the review, including how many reviewers screened each record and each report retrieved, whether they worked independently, and if applicable, details of automation tools used in the process.	Methods, Section 2.4 (line [28]): Two reviewers independently screened titles/abstracts and full texts. Discrepancies resolved by discussion and consensus.
Data collection process	9	Specify the methods used to collect data from reports, including how many reviewers collected data from each report, whether they worked independently, any processes for obtaining or confirming data from study investigators, and if applicable, details of automation tools used in the process.	Methods, Section 2.5 (lines [30]–[31]): Data extracted independently by two reviewers using

PRISMA 2020 Checklist

Section and Topic	Item #	Checklist item	Location where item is reported
			a standardized form. Discrepancies resolved by referring to original study and reaching consensus.
Data items	10a	List and define all outcomes for which data were sought. Specify whether all results that were compatible with each outcome domain in each study were sought (e.g. for all measures, time points, analyses), and if not, the methods used to decide which results to collect.	Methods, Section 2.2 (line [23]): Primary: SE ±0.50 D, CYL ≤0.50 D. Secondary: UDVA ≥20/20, CDVA loss ≥1 line, SEQ R^2, CYL R^2, TIA, SIA, axis alignment ±5°, HOAs (total, spherical, coma). All time points sought.
	10b	List and define all other variables for which data were sought (e.g. participant and intervention characteristics, funding sources). Describe any assumptions made about any missing or unclear information.	Methods, Section 2.5 (line [30]): Study characteristics (author, year, PMID, journal, country, design, sample size, follow-up), patient demographics (age, sex, preoperative refraction), surgical parameters (laser settings, nomogram, cyclotorsion compensation).
Study risk of bias assessment	11	Specify the methods used to assess risk of bias in the included studies, including details of the tool(s) used, how many reviewers assessed each study and whether they worked independently, and if applicable, details of automation tools used in the process.	Methods, Section 2.6 (line [33]): ROB2 for RCTs, ROBINS-I for non-randomized studies. Two reviewers independently assessed each study.
Effect measures	12	Specify for each outcome the effect measure(s) (e.g. risk ratio, mean difference) used in the synthesis or presentation of results.	Methods, Section

Section and Topic	Item #	Checklist item	Location where item is reported
			2.7.1 (line [35]): Risk ratio (RR) using Mantel-Haenszel random-effects for binary outcomes. Mean difference (MD) for continuous outcomes using DerSimonian-Laird random-effects.
Synthesis methods	13a	Describe the processes used to decide which studies were eligible for each synthesis (e.g. tabulating the study intervention characteristics and comparing against the planned groups for each synthesis (item #5)).	Methods, Section 2.2: Studies grouped by outcome type (primary: predictability; secondary: astigmatism, visual acuity, safety, correlation, HOAs). Only comparative studies included.
	13b	Describe any methods required to prepare the data for presentation or synthesis, such as handling of missing summary statistics, or data conversions.	Methods, Section 2.7.1 (line [35]): For paired data (SEQ R^2, CYL R^2), paired t-test was performed using the difference between VISUMAX 800 and VISUMAX 500 values within each study.
	13c	Describe any methods used to tabulate or visually display results of individual studies and syntheses.	Methods, Section 2.7.1 (line [35]) and Results: Forest plots for all meta-analyzed outcomes. Box plots for R^2 comparisons. Figures 3–6.
	13d	Describe any methods used to synthesize results and provide a rationale for the choice(s). If meta-analysis was performed, describe	Methods, Section

Section and Topic	Item #	Checklist item	Location where item is reported
		the model(s), method(s) to identify the presence and extent of statistical heterogeneity, and software package(s) used.	2.7: Random-effects model (DerSimonian-Laird). Heterogeneity assessed via I^2, Cochran's Q. Software: R 4.5.1, meta (7.0-0), metafor (4.0-0), robvis (0.3.0) packages.
	13e	Describe any methods used to explore possible causes of heterogeneity among study results (e.g. subgroup analysis, meta-regression).	Methods, Section 2.7.3 (line [37]): Leave-one-out sensitivity analysis for SE ±0.50 D.
	13f	Describe any sensitivity analyses conducted to assess robustness of the synthesized results.	Methods, Section 2.7.3 (line [37]) and Results Section 3.7 (line [195]): Leave-one-out sensitivity analysis for primary outcome (SE ±0.50 D). Table 3.
Reporting bias assessment	14	Describe any methods used to assess risk of bias due to missing results in a synthesis (arising from reporting biases).	Methods, Section 2.7.4 (line [38]): Funnel plots (visual), Egger's regression test, Begg's rank correlation test, trim-and-fill method. $P<0.10$ threshold.
Certainty assessment	15	Describe any methods used to assess certainty (or confidence) in the body of evidence for an outcome.	Methods, Section 2.7: No formal GRADE assessment described. I^2 and Q statistic used for heterogeneity appraisal.

Section and Topic	Item #	Checklist item	Location where item is reported
**RESULTS**			
Study selection	16a	Describe the results of the search and selection process, from the number of records identified in the search to the number of studies included in the review, ideally using a flow diagram.	Results, Section 3.1 (line [42]) and Figure 1: PRISMA flow diagram. 11 records identified, 11 screened, 9 included.
	16b	Cite studies that might appear to meet the inclusion criteria, but which were excluded, and explain why they were excluded.	Results, Section 3.1 (line [42]): Two studies excluded: Sekundo 2025 [22] (lacked VISUMAX 500 comparator), Lee 2025 [23] (single-arm case series). Reasons documented.
Study characteristics	17	Cite each included study and present its characteristics.	Results, Section 3.2 (lines [45]–[136]) and Table 1: Characteristics of all 9 included studies presented (author, year, PMID, journal, country, design, sample size, follow-up).
Risk of bias in studies	18	Present assessments of risk of bias for each included study.	Results, Section 3.3 (line [138]) and Figure 2: ROBINS-I/ROB2 assessments. 6 studies "some concerns", 3 studies "high" risk. Traffic light and summary plots.
Results of individual studies	19	For all outcomes, present, for each study: (a) summary statistics for each group (where appropriate) and (b) an effect estimate and its precision (e.g. confidence/credible interval), ideally using structured tables or plots.	Results, Sections 3.4–3.5 (lines [141]–

Section and Topic	Item #	Checklist item	Location where item is reported
			[159]): Forest plots for each outcome show study-level summary statistics and effect estimates. Figures 3–6.
Results of syntheses	20a	For each synthesis, briefly summarise the characteristics and risk of bias among contributing studies.	Results, Section 3.3 (line [138]) and Section 3.4–3.5: Risk of bias of contributing studies summarized in Figure 2. Study characteristics in Table 1.
	20b	Present results of all statistical syntheses conducted. If meta-analysis was done, present for each the summary estimate and its precision (e.g. confidence/credible interval) and measures of statistical heterogeneity. If comparing groups, describe the direction of the effect.	Results, Sections 3.4–3.5: Summary estimates (RR, MD) with 95% CIs, P-values, I^2 for each outcome. Figures 3–6.
	20c	Present results of all investigations of possible causes of heterogeneity among study results.	Results, Sections 3.4–3.5: I^2 and Cochran's Q reported for each analysis. Discussion of potential sources (Varman 2024 outlier). No formal subgroup analysis performed.
	20d	Present results of all sensitivity analyses conducted to assess the robustness of the synthesized results.	Results, Section 3.7 (line [195]): Leave-one-out sensitivity analysis. Table 3 shows RR, 95% CI, P-value after each exclusion.

PRISMA 2020 Checklist

Section and Topic	Item #	Checklist item	Location where item is reported
Reporting biases	21	Present assessments of risk of bias due to missing results (arising from reporting biases) for each synthesis assessed.	Results, Section 3.6 (lines [161]–[162]) and Table 2: Funnel plots (Figure S1), Egger's test, Begg's test, trim-and-fill results. No significant publication bias detected.
Certainty of evidence	22	Present assessments of certainty (or confidence) in the body of evidence for each outcome assessed.	Results, Sections 3.4–3.7: I^2 values, sensitivity analysis, and publication bias tests reported. No formal GRADE assessment.
**DISCUSSION**			
Discussion	23a	Provide a general interpretation of the results in the context of other evidence.	Discussion, paragraph [264]–[269]: Results contextualized with existing literature. Comparison to individual study findings.
	23b	Discuss any limitations of the evidence included in the review.	Discussion, paragraph [270]: Limitations discussed — absence of RCTs, confounding, heterogeneity, short follow-up, publication bias possibility.
	23c	Discuss any limitations of the review processes used.	Discussion, paragraph [270]: Limitations of review processes — pre-

PRISMA 2020 Checklist

Section and Topic	Item #	Checklist item	Location where item is reported
			registered protocol, PRISMA methodology applied, but limited to comparative studies with extractable numerical data.
	23d	Discuss implications of the results for practice, policy, and future research.	Discussion, paragraphs [265]–[269]: Implications for clinical practice (VISUMAX 800 axis alignment advantage, shorter procedure time) and future research (need for RCTs, standardized outcomes, longer follow-up).
**OTHER INFORMATION**			
Registration and protocol	24a	Provide registration information for the review, including register name and registration number, or state that the review was not registered.	Methods, Section 2.1 (line [17]): "Pre-registered PROSPERO: CRD420261407686"
	24b	Indicate where the review protocol can be accessed, or state that a protocol was not prepared.	Methods, Section 2.1 (line [17]): Protocol registered on PROSPERO (CRD420261407686).
	24c	Describe and explain any amendments to information provided at registration or in the protocol.	Methods, Section 2.1: No amendments to protocol reported.
Support	25	Describe sources of financial or non-financial support for the review, and the role of the funders or sponsors in the review.	Discussion, line [281]: "Funding: Not applicable"

PRISMA 2020 Checklist

Section and Topic	Item #	Checklist item	Location where item is reported
Competing interests	26	Declare any competing interests of review authors.	Discussion, lines [276]–[277]: "There are no competing interests among authors"
Availability of data, code and other materials	27	Report which of the following are publicly available and where they can be found: template data collection forms; data extracted from included studies; data used for all analyses; analytic code; any other materials used in the review.	Discussion, lines [282]–[283]: "The datasets used in the current study are available from the corresponding author on reasonable request."

From: Page MJ, McKenzie JE, Bossuyt PM, Boutron I, Hoffmann TC, Mulrow CD, et al. The PRISMA 2020 statement: an updated guideline for reporting systematic reviews. BMJ 2021;372:n71. doi: 10.1136/bmj.n71. This work is licensed under CC BY 4.0. To view a copy of this license, visit https://creativecommons.org/licenses/by/4.0/
