# Supplementary figures and images for: Comparison of VISUMAX 800 and VISUMAX 500 Femtosecond Laser Systems for Myopia: A Systematic Review and Meta-Analysis
Source: J Clin Med. 2026 Jul 14;15(14):5517. doi: 10.3390/jcm15145517 (PMC13412861; doi:10.3390/jcm15145517)

A

**Funnel Plot: SE  $\pm 0.50$  D  
with Pseudo 95% CI**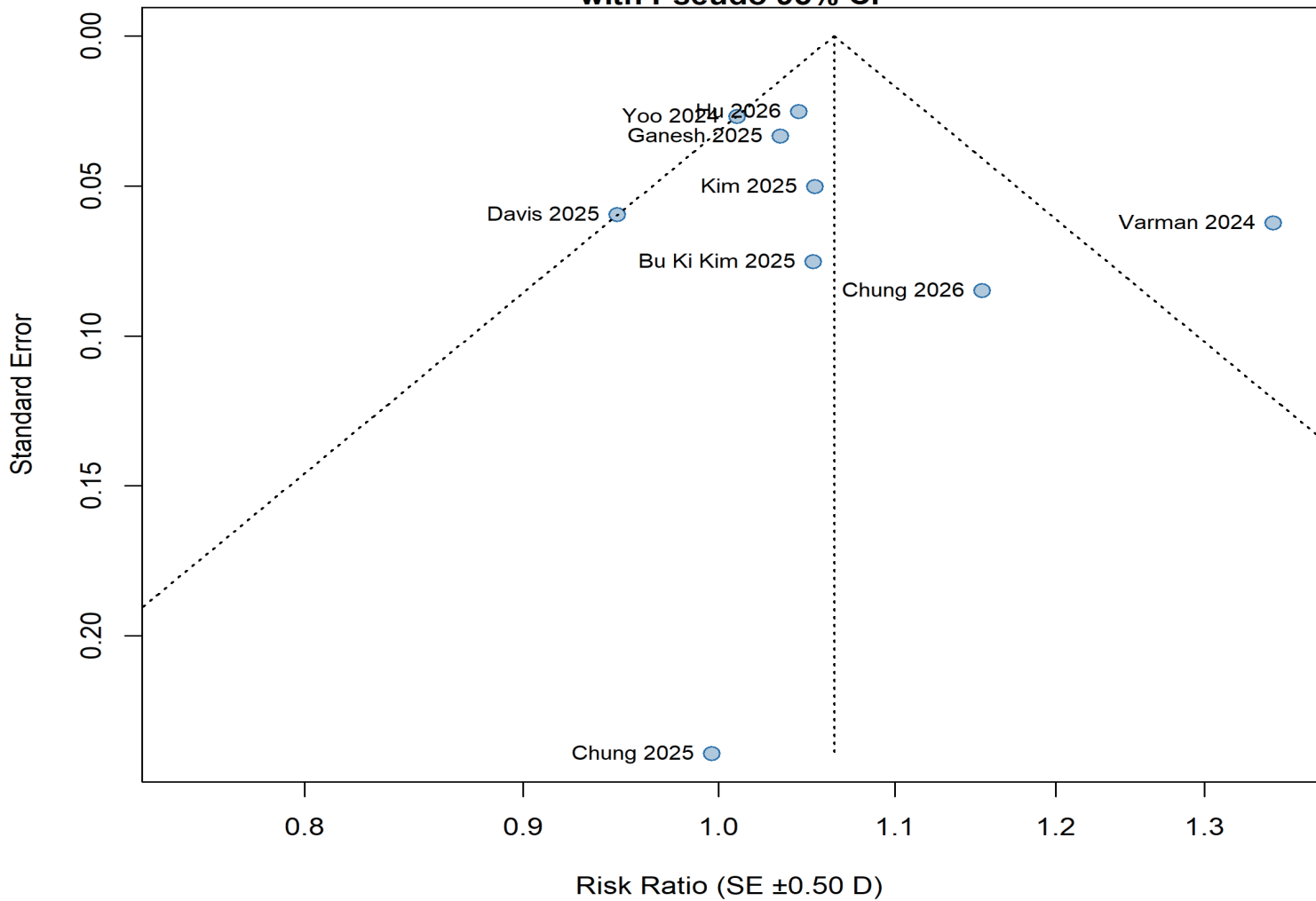

B

**Funnel Plot: CYL  $\leq 0.50$  D  
with Pseudo 95% CI**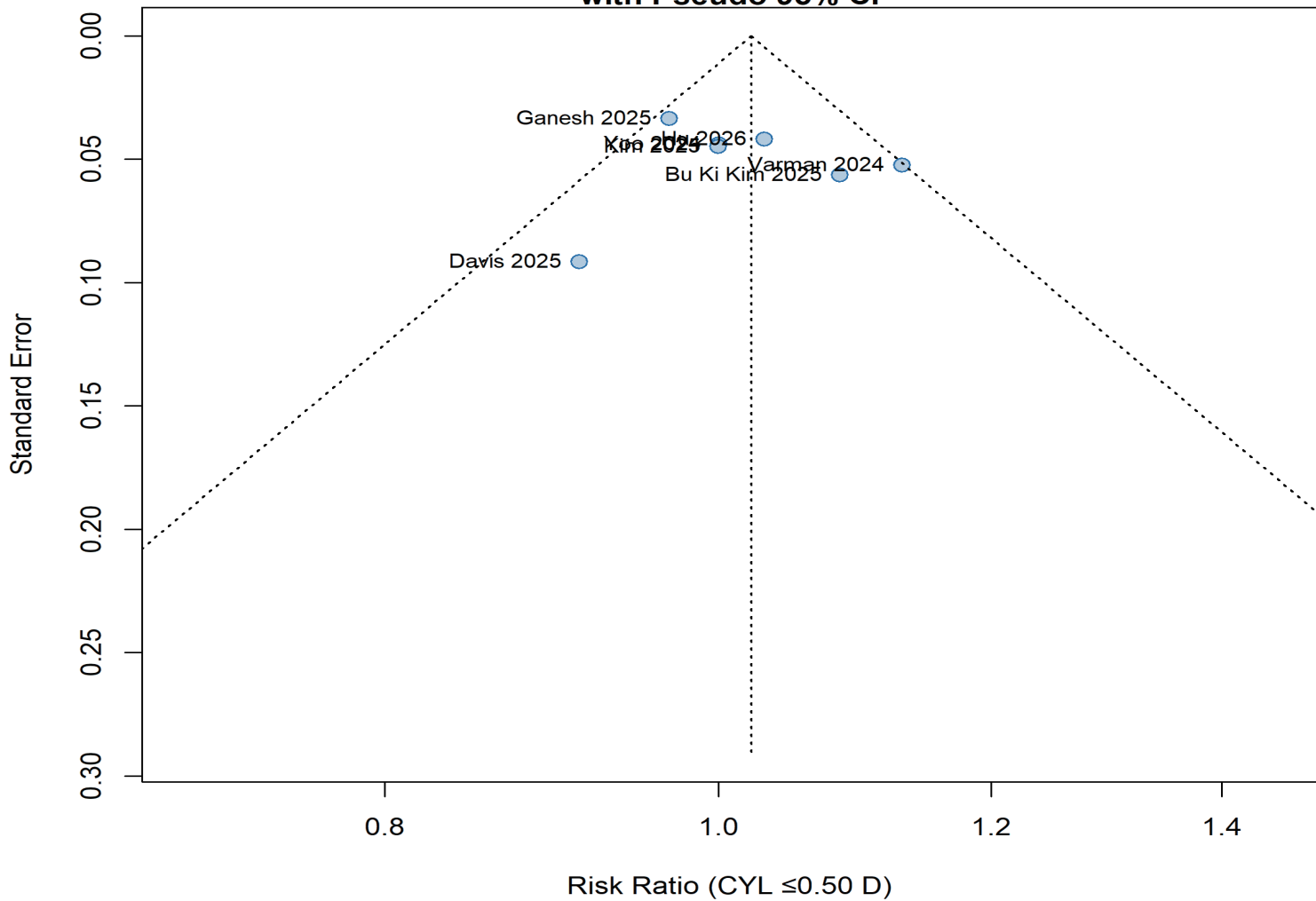

Supplement: Supplementary file 1 [file jcm-15-05517-s001.zip › Figure S1.pdf]
